# Role of mutans streptococci, acid tolerant bacteria and oral *Candida* species in predicting the onset of early childhood caries

**DOI:** 10.3389/fdmed.2023.991746

**Published:** 2023-03-03

**Authors:** Alissa Villhauer, Min Zhu, Wei Shi, Xian Jin Xie, Pamella Hughes, Amy Lesch, Karin Weber-Gasparoni, Justine Kolker, David Drake, Jeffrey A. Banas

**Affiliations:** ^1^ Iowa Institute for Oral Health Research, University of Iowa College of Dentistry and Dental Clinics, Iowa City, IA, United States; ^2^ Division of Biostatistics and Computational Biology, University of Iowa College of Dentistry and Dental Clinics, Iowa City, IA, United States; ^3^ Department of Pediatric Dentistry, University of Iowa College of Dentistry and Dental Clinics, Iowa City, IA, United States; ^4^ Department of Operative Dentistry, University of Iowa College of Dentistry and Dental Clinics, Iowa City, IA, United States

**Keywords:** mutans streptococci (MS), oral candida, co-colonization, acid tolerant bacteria, early childhood caries (ECC), caries risk assessment

## Abstract

**Aim:**

Early childhood caries is the most common chronic infectious disease in children in the United States. This study, which is part of a larger, longitudinal study exploring oral microbiological components of caries development in children, reports on the impact of total mutans streptococci (MS), total acid tolerant bacteria and *Candida* species on the development of dental caries in a subset of these children. Of particular interest was the relationship between caries development and co-colonization of mutans streptococci and *Candida* species.

**Methods:**

Children between the ages of 12 and 47 months displaying no evidence of dental caries were recruited for a longitudinal study (*n* = 130). Twelve age- and gender-matched pairs were selected. In each pair, one child developed caries during the study, and one did not. Whole mouth plaque samples were collected by swab at baseline and every 6 months thereafter for a duration of 18 months and spiral plated for microbial counts (CFU/ml). Cut-offs based on percent of total cultivable flora were designated for all microbial measures. A scoring system designated by the Plaque Microbial Index (PMI) was developed for use in statistical analyses to assess potential predictive factors for caries risk assessment.

**Results:**

Children who developed caries were significantly more likely to harbor higher percentages of acid tolerant bacteria (*p* = 0.003), MS (*p* < 0.001) and have *Candida* species present (*p* < 0.001) at ≥1 visit leading up to caries onset. Mean PMI scores derived from the aforementioned microbial measures, were higher for caries-active children than for caries-free children (*p* = 0.000147). Co-colonization of MS and *Candida* species was significantly associated with caries development (*p* < 0.001) and detection of both at the same visit had a 100% positive predictive value and 60% negative predictive value for caries development.

**Conclusion:**

In children who developed caries, there was a statistically significant association with the percent of total flora that was acid tolerant, the percent of MS, the presence of *Candida* and co-colonization of MS and *Candida* species. Combining these microbial measures into PMI scores further delineated children who developed caries from those who remained caries-free. These microbiological measures show potential as predictive factors and risk assessment tools for caries development.

## 1. Introduction

Early childhood caries (ECC) impacts more children in the United States than any other chronic infectious disease (1). As a contributing factor to overall health and quality of life, poor oral health in children has a far-reaching impact. Early childhood caries is a multifactorial disease involving the complex interaction of factors such as environmental influences, socio-economic status, oral hygiene practices, host susceptibility, diet and host microbiome composition, especially the acquisition of cariogenic bacterial species (2–4).

The composition of the oral microbiome has been the focus of much ECC research. The Ecological Plaque Hypothesis theorizes that there is a shift in oral flora from a state of homeostasis to one of dysbiosis that eventually leads to the development of dental caries (5, 6). Multiple studies have also shown that there are specific cariogenic bacterial species that are commonly present in the microbiota of the oral cavity of carious individuals. Mutans streptococci (MS) are acid-producing primary colonizers that have frequently been cited as significant etiological agents of dental caries (4, 7–9). Members of the fungal genus *Candida* have also been linked to ECC (10–12). There has been an increased focus on the potential synergistic effect of mutans streptococci and *Candida* species co-colonization in plaque biofilms (13–16). Strong acidogens, such as the mutans streptococci, can lower the pH of the surrounding plaque in conjunction with its prolific synthesis of biofilm-promoting extracellular polysaccharide, contributing to a shift in the microbiota of the area and creating more favorable conditions for additional acidogenic and aciduric bacterial species, ultimately leading to demineralization of tooth enamel (12, 17, 18).

This study was part of a larger, ongoing longitudinal study examining multiple microbiological elements in children leading up to the onset of ECC. The purpose of this study is to evaluate the presence and burden of three targeted microbial measures before and at the time of initial caries onset with the goal of working toward a potential means of assessing caries risk. The selected targets for these analyses were mutans streptococci, acid tolerant bacteria (bacteria that will grow on Brain Heart Infusion agar adjusted to a pH of 5.0) and *Candida* species. Here, we investigate the differences between the microbial profiles of children who developed caries over the course of the study and those who did not, in a subset of the total study population.

## 2. Materials and methods

### 2.1. Study population, recruitment and consent

Children between the ages of 12 and 47 months with at least one erupted tooth, no evidence of current or past caries, without any serious systemic disease (as affirmed by parents or legal guardian) and with no use of any antibiotics within the previous 3 months were recruited from the University of Iowa College of Dentistry and Dental Clinics Department of Pediatric Dentistry for a longitudinal study. Participation in the study involved caries examinations and collection of pooled plaque samples at four time points (baseline, 6 months, 12 months, and 18 months ±30 days). This study was reviewed and approved by the University of Iowa Institutional Review Board (IRB number 201805812). Parents or legal guardians of children scheduled for dental examinations who appeared to meet study requirements were contacted by the study coordinator and asked if they would like to participate. Consent was obtained from the parents or legal guardians who chose to enroll their children in the study.

### 2.2. Clinical examination

At the baseline visit, following the consent procedure, a trained and calibrated dentist performed a dental examination using the knee-to-knee examination technique (19) on each child to confirm the absence of caries. If any carious lesions were detected, the child was excluded from the study and the family was provided with a report on the examination and recommendations for follow-up care. For children who met the study criteria, at each follow-up visit, repeat dental examinations were performed to determine if any decay had developed between visits. All examinations were scored using the d_1_d_2·3_ caries criteria developed by Warren et al. (20). Two dentists were calibrated to perform examinations and collect samples for this study.

### 2.3. Sample collection and processing

Prior to the caries examination, the dentist performing the examination collected a pooled plaque sample by swabbing the buccal, lingual, and occlusal surfaces of all erupted teeth with a sterile cotton swab. Plaque samples were placed in 1 ml of half-strength Trypticase Soy Broth supplemented with 2.5% Yeast extract (TSB-YE) (Becton, Dickinson and Company, Sparks, MD, United States), and transported to the microbiology labs within the Iowa Institute for Oral Health Research at the University of Iowa College of Dentistry and Dental Clinics.

Upon delivery, samples were vortexed (15 s, highest setting) and placed in a sonicating water bath for one minute to assure homogeneous suspensions of plaque microbiota. Samples were serially diluted and spiral plated for determination of the following counts (CFU/ml) using standard spiral plating methodology (Whitley Automatic Spiral Plater, Microbiology International, Frederick, MD, United States):

1. Total cultivable flora—CDC blood agar (Remel, Lenexa KS).
2. Total acid tolerant bacteria—Brain Heart Infusion (BHI) agar (Becton, Dickinson and Company, Sparks, MD, United States) adjusted to pH 5.0 by addition of 37% hydrochloric acid to the media until it reached a pH of 5.0, followed by autoclaving and plate preparation.
3. Total mutans streptococci—Modified SB20 agar prepared as detailed by Saravia et al. (21).
4. Total *Candida*—HardyCHROM *Candida* agar (Hardy Diagnostics, Santa Maria, CA)

CDC blood agar and BHI agar were incubated 48 h anaerobically at 37°C, followed by 24 h incubation at 37°C, 5% CO_2_. The SB20M plates were incubated for 48 h at 37°C, 5% CO_2_. *Candida* plates were incubated aerobically at 37°C for 48 h.

### 2.4. Selection of subjects for analyses

For this initial exploration of microbial profiles, children were selected only from those who had completed their final (18-month) visit. Of those subjects, each child who had developed caries over the course of the study was age- and gender-matched with a child that did not develop caries. This resulted in twelve pairs for these analyses. This study is focused on microbiological profiles that may be predictive of caries development, so all visits leading up to and including the visit where caries activity was first detected were analyzed. For example, if caries was detected in a child at the 12 month visit, the baseline, 6 month, and 12 month visits from that child and the matched caries free child were included in the analyses.

### 2.5. Data collection, scoring and statistical methods

Total cultivable flora, total acid tolerant bacteria, total mutans streptococci, and total *Candida* counts were recorded. Percent of total flora was calculated for acid tolerant, mutans streptococci, and *Candida*. A cut-off was determined for each microbial measure based on the percent of total flora. A binary scoring system was used to classify each category at each visit. Any visit with a percentage at or above the cut-off was considered a positive test and received a score of one, while any visit below the cut-off received a score of zero. The cut-offs were set as follows: acid tolerant bacteria ≥1% total flora, MS ≥0.1% of total flora and, due to the low percentages present, *Candida* species were recorded as present/absent (1/0) for statistical analyses. Additionally, a total score designated the Plaque Microbial Index (PMI) was assigned for each visit based upon the individual category scores (i.e., mutans streptococci present above cutoff (1) and *Candida* present (1) would have a visit score of 2). After collecting data for all visits, each child was assigned an Overall Plaque Microbial Index (OPMI) based on what was seen across all visits. Potential scores ranged from zero (never met any criteria) to three (all criteria met on a cumulative basis). Those children who received OPMI scores of 2 or 3 were above the cutoff for multiple microbial measures at a minimum of 1 visit though not necessarily at the same visit (i.e., Child with acid tolerant present at baseline, acid tolerant + MS at 6 months and MS + *Candida* at 12 months would receive a score of 3 because all three microbial measures were detected at some point during the study). For the scope of this paper, results indicating that a microbial measure was present means it was detected at levels above the designated cut-off.

Plaque Microbial Index, Overall Plaque Microbial Index, mean PMI over time, and MS/*Candida* co-colonization data were evaluated utilizing Students' *t*-test. Linear Mixed Model (repeat measurement) was performed before combining visit data for scoring purposes and was consistent with Students' *t*-test results. Fisher's exact test was used in binary measure analyses while a generalized linear model (binomial, logistic function) was performed to evaluate the correlation of dependent variables (binary measurements) with predictors of caries status and time.

## 3. Results

Samples were collected every six months throughout the study. For children who developed caries by the end of the study, the first visit where caries was diagnosed was determined and all samples from visits leading up to and including that time point were analyzed. Due to children developing caries at varying timepoints, not all pairs had results from the same number of visits. However, because each caries-active (CA) child was age- and gender-matched with a caries-free (CF) child who had also completed the study (*n* = 12 pairs), we were able to analyze an equivalent number of visits for each group (CA = 33, CF = 33). Sampling times were designated by the number of months prior to the diagnosis of caries at which the sample was taken. The designations were: minus 18 months, minus 12 months, minus 6 months, and Time 0 (caries diagnosed).

### 3.1. Acid tolerant, mutans streptococci and *Candida* microbial burden

An initial analysis of the presence of each individual microbial measure across all visits showed that children who developed caries over the course of the study were significantly more likely to display acid tolerant bacteria comprising ≥1% of their total cultivable flora (*p* = 0.003), MS comprising ≥0.1% of total flora (*p* < 0.001) and have *Candida* present (*p* < 0.001) at ≥1 visit throughout the study. When evaluating individual sampling times, with the exception of acid tolerant (*p* = 0.012) and MS (*p* = 0.036) at Time 0, no significant differences were found at the defined time points for acid tolerant (*p* = 0.23), MS (0.61) or *Candida* (*p *= 0.50) (Table 1). Supplementary materials have been provided with raw microbial counts data (CFU/ml) for each visit and mean counts across all visits (Supplementary Table S1).

**Table 1:** Microbiological burden and association with caries development across time.

Microbial measure	Visit (time before caries onset)	*N* ^a^	Percent positive visits (Binary measure = 1)		*p*-value*
			Caries Active	Caries Free	
Acid tolerant bacteria ≥1% of total cultivable flora (Binary measure = 1)	Minus 18 months	6	50%	50%	1.00
	Minus 12 months	7	86%	29%	0.103
	Minus 6 Months	8	75%	50%	0.608
	Time 0	12	75%	17%	0.012*
	All visits	33	73%	33%	0.003*
Mutans streptococci ≥0.1% of total cultivable flora (Binary measure = 1)	Minus 18 months	6	50%	17%	0.545
	Minus 12 months	7	57%	0%	0.07
	Minus 6 Months	8	88%	38%	0.119
	Time 0	12	67%	17%	0.036*
	All visits	33	67%	18%	<0.001*
*Candida* species present (Binary measure = 1)	Minus 18 months	6	50%	0%	0.182
	Minus 12 months	7	43%	14%	0.559
	Minus 6 Months	8	38%	0%	0.200
	Time 0	12	33%	0%	0.093
	All visits	33	39%	3%	<0.001*

^a^ *N* = number of pairs; eight pairs is equal to 16 children (8 CA, 8 CF).

* Significance probability (*p*-value) determined with generalized linear modeling (binomial, logistic function) of association between caries status and percent of visits with a positive test (binary value = 1) for each microbial measure. Results with a *p*-value of ≤0.05 were significant.

To determine the impact of considering the microbial measures in combination, a Plaque Microbial Index (PMI) was created to assess individual visits or cumulative visits (Overall Plaque Microbial Index; OPMI) for each subject. Analyses of the mean PMI score per visit showed that CA children had a higher average score than CF children (*p* = 0.000147) (Table 2). The mean OPMI score, which reflected the microbial measures that were present above the designated cut-off at ≥1 visit, was also higher in the children who developed caries (*p* = 0.009) (Table 2). The mean PMI score per visit at each individual timepoint ahead of caries development is significantly different between groups at all visits except 18 months before caries (Table 3).

**Table 2:** Mean plaque microbial Index (PMI) per visit and mean overall plaque microbial Index (OPMI) and their association with caries status.

Variable	Caries status	*N*	Mean	Standard Deviation	*p*-value*
Mean PMI per visit	Caries Active	12	1.76	0.78	0.000147*
	Caries Free	12	0.53	0.52	
Mean OPMI	Caries Active	12	2.17	0.83	0.009*
	Caries Free	12	1.08	0.996	

* Significance probability (*p*-value) by Students’ *t*-test of association between mean PMI per visit and caries status, as well as OPMI and caries status. Both results were statistically significant.

**Table 3:** Mean plaque microbial index (PMI) per visit at each designated time point ahead of caries diagnosis.

Visit (time before caries onset)	Caries status	*N*	Mean	Standard Deviation	*p*-value*
Minus 18 months	Caries Active	6	1.50	1.049	0.16
	Caries Free	6	0.67	0.816	
Minus 12 months	Caries Active	7	1.86	0.900	0.008*
	Caries Free	7	0.43	0.787	
Minus 6 months	Caries Active	8	2.00	0.926	0.023*
	Caries Free	8	0.88	0.835	
Time 0	Caries Active	12	1.75	0.754	<0.001*
	Caries Free	12	0.33	0.651	

* Significance probability (*p*-value) by Students’ *t*-test of association between mean visit score and caries status. Results with *p*-values ≤0.05 were significant.

### 3.2. Mutans streptococci and *Candida* co-colonization

Co-colonization of MS and *Candida* was examined under three conditions and was significantly associated with the development of caries in all circumstances (Table 4). Broadly, we determined the number of visits where MS and/or *Candida* were detected in all CA and CF children. The percentage of positive visits was higher in CA children (*p* < 0.001). When narrowing the parameters to the number of visits with MS and/or *Candida* in only children who harbored both at ≥1 visit (not necessarily at the same time), the percentage of positive visits was again higher in children with caries (*p* < 0.001). We further limited the analyses to the number of visits with MS and *Candida* present at the same time. This again resulted in significantly more positive visits in CA children (*p* = 0.002). In this subset, detection of MS and *Candida* at the same visit (positive test) was 100% predictive of caries development. Four children (33%) in the CA group displayed co-colonization. Of the remaining 20 children, 12 remained CF for the duration of the study, which resulted in a predictive value of 60% for a negative test (no co-colonization).

**Table 4:** Co-colonization of MS and *Candida* and its association with caries.

Colonization parameter	Percent of visits (*n* = 33)^a^ that fulfill colonization parameter		*p*-value*
	Caries active	Caries free	
Visits with MS and/or *Candida* (all children)	79% (*n* = 26)	21% (*n* = 7)	<0.001*
Visits with MS and/or *Candida* (only children with each detected at ≥1 visit)	45% (*n* = 15)	6% (*n* = 2)	<0.001*
Visits with MS and *Candida* (detected at the same visit)	27% (*n* = 9)	0% (*n* = 0)	0.002*

^a^ Thirty-three visits per group (33 visits from CA children, 33 from CF children).

* Significance probability (*p*-value) by Students’ *t*-test of association between mutans streptococci and *Candida* co-colonization parameters and caries status. All results were statistically significant.

## 4. Discussion

In this study, we evaluated the potential efficacy of utilizing microbiological measures, individually or in combination, as predictors of ECC risk in a cohort of young children. We also explored the viability of using MS/*Candida* co-colonization as an accurate marker for increased risk of future caries development. Our results confirmed that both approaches showed significant differences between CA and CF children leading up to caries diagnosis, with MS + *Candida* presence at the same sampling time being 100% predictive of ECC development in this subset of children. The longitudinal nature of the study provided an opportunity to expand current knowledge of microbial interactions occurring in the oral cavities of young children prior to clinical detection of ECC. This is critical because microbiome changes that are significant to the shift from microbial balance to dysbiosis do not only occur during caries development, but in the time leading up to its clinical diagnosis (22).

Multiple studies and systematic reviews have confirmed a correlation between mutans streptococci and caries initiation and progression (4, 8, 9, 22–25). Additional acidogenic/aciduric species that have frequently been found to be associated with ECC include non-mutans streptococci, lactobacilli, *Actinomyces* and *Candida* species (7, 23–26). While MS and other low pH species have consistently been cited as etiological agents in dental caries, as observed in our study, they are not universally detected in all caries active individuals. At the same time, presence of a cariogenic species does not automatically result in caries development. Twenty-five percent (3/12) of the CA children in this study did not harbor MS above the designated cut-off percentage at any time point while 42% (5/12) of CF children did have MS at levels above the cut-off at ≥1 visit. Additionally, even with the prevalence of data supporting the contribution of low pH bacteria to caries, we still observed one child in the CA group that never met the criteria for percent of acid tolerant bacteria at any visit leading up to caries diagnosis and seven CF children (58%) who did.

The approach chosen for this study was to set a cut-off value for microbial measures—acid tolerant bacteria (≥1% total flora), mutans streptococci (≥0.1% of total flora) and *Candida* species (present/absent). Higher percentages indicated higher burden of potentially cariogenic microbes detected. Cut-offs for acid tolerant bacteria and MS were determined by looking at the counts for all subjects at all visits for each bacterial category. Since this was a new approach, there was no existing threshold to use. When setting cut-offs, we looked for a consistent point where there was a noticeable drop in percent of total cultivable bacteria and then looked at the categorization (CA or CF) of each visit above and below the cut-off to see what percentage of each were above and below the cut-off. For acid tolerant bacteria, utilizing the ≥1% of total cultivable bacteria cut-off, almost 70% of the visits above the cut-off were from CA children while a little over 70% of the visits below the cut-off were from CF children. With the MS, cut-off set at ≥0.1% of total cultivable bacteria, just over 75% of visits above the cut-off were from CA children and 70% of visits below the cut-off were from CF children. The study team concluded that these were reasonable cut-offs based on the data available in this study and results from statistical analyses validated the cut-offs selected for this study. Verification of the cut-offs with another independent dataset will be essential before considering the adoption of the cut-offs for clinical use. Percent of total cultivable flora, as opposed to CFU/ml counts, was selected for analysis in this study. This is due to the fact that this study was focused on very young children, so there is not only the natural variation in CFU/ml counts one would observe in any study, but also variation in number of erupted teeth (therefore variation in amount of tooth surface available for colonization). We ultimately decided that focusing on percentage instead of total CFU/ml would be the best approach to minimize the impact of these differences.

Each microbial measure was scored as zero (below cut-off) or 1 (above cut-off) and the three scores were combined at each visit and designated as the Plaque Microbial Index (PMI) for that visit. We also evaluated which microbial measures were present across all visits and assigned each subject an Overall Plaque Microbial Index (OPMI) score reflecting what was detected above cut-off values throughout the time leading up to caries. As stated previously, scores ranged from zero to three. Looking at the final data for CA and CF children, based on the scoring system we developed, a subject with an OPMI of zero or 1 would be considered at low risk of developing caries. There was an exception to this categorization. If the score was 1 and the percent of total flora in that one category was much higher than the cut-off (i.e., CA child whose acid tolerant count was 13% of total flora) or they only had *Candida* present (almost exclusively associated with CA children), then that subject would be considered to have a higher risk. Sixty percent (8/12) of CF children had a score of zero or 1. No CA children had a score of zero. Three children with caries had a score of 1, but all were at high percentages in their respective microbial measure. A subject with a score of 2 would be considered at moderate risk of caries development and a score of 3 would be considered high risk. In CA children, 42% (5/12) had an OPMI of 3 and an additional 33% (4/12) had a score of 2. One child in the caries free group had an overall score of 3. While they did not develop caries within the timeframe of the study, we would consider them as having a high risk for caries in the future.

As seen in Table 2, mean PMI and mean OPMI are significantly different between CA and CF children. Scores are significantly higher in the caries active group. When looking at individual time points ahead of caries diagnosis (Table 3), significant differences were observed between groups up to 12 months pre-caries onset. In this subset, the lack of significance in the minus 18-month samples may be due to the smaller sample size or the differences between groups are not as strong that far in advance of caries development. Even though the minus 18-month sample was not statistically significant, when all visits over the course of the study were considered together, there was a statistically significant difference between the CA and CF groups, underscoring the importance of the longitudinal aspect of this study. This also reinforces the importance of repeat visits for patient caries risk assessment (CRA).

In addition to the PMI and OPMI scores, we evaluated co-colonization of MS and *Candida* species. A growing body of research has indicated that *Candida* species are frequently detected in the dental plaque of ECC patients (14, 27–29). There has also been a focus on the synergistic relationship between MS and *Candida* species (10, 13, 14, 16, 30), so the interaction of these two groups was of interest in this study. Biofilms formed with both species present are more robust, more complex and more resilient than those containing only one of the species (16, 31). Research indicates that *Candida* can adhere to the biofilm by utilizing glucan binding sites and attaching to glucans produced by mutans streptococci forming a strong framework for additional aciduric and potentially cariogenic bacteria to attach (30, 32). Co-colonization leads to a highly acidic plaque environment and rapid onset of severe disease (16, 29). Seven children in our study population had *Candida* detected at ≥1 visit. Six of the seven were in the CA group. The remaining caries free child was the single CF subject with an OPMI score of three. As Table 4 indicates, MS/*Candida* co-colonization was significantly associated with caries activity, even when they were detected at separate visits. When detected at the same visit, the subject eventually developed caries 100% of the time.

No matter how compelling the evidence for the role of MS, *Candida* and other acidogenic/aciduric bacteria in ECC, the oral microbiome is dynamic, especially in young children. Our data show that screening for a single microbiological factor or only at one time point is not as effective as a combined approach over time to assess future caries risk. This is in agreement with conclusions drawn from comprehensive reviews that have shown multifactorial analyses to be of more value in CRA models and caries prediction (33–36). CRA models generally include screening for a range of factors, including socio-economic status, oral hygiene habits, fluoride exposure, sugar consumption, caregiver's education level, ethnicity, access to care and other influences that have been shown to be associated with oral health and disease (33, 37). Additionally, they may include screening for MS due to the high association with dental caries (33–35). Predictive modeling that includes microbiological factors has been explored through screening for specific species and computer modeling based on oral microbiome sampling (22, 36, 38, 39). Full oral microbiome studies and reviews have noted more complex combinations of microbial and metabolic factors associated with oral health and diseases (22, 40–42). While vital in adding information to the complex picture of caries development and enhancing new predictive models, our goal was a simpler approach that could potentially be developed into a point-of-care screening tool to be combined with existing CRA methods.

In their Policy on Early Childhood Caries, the American Academy of Pediatric Dentistry reaffirmed the significant impact of ECC on the health of young children and the importance of incorporating early assessment and preventive measures into clinical care practices (43). In evaluating multiple systematic reviews of existing CRA models, there was an acknowledged need for accurate assessment methods, especially for young children, but also a recognized lack of evidence-based studies to verify the accuracy of each model (33, 34, 37). Because existing socio-demographic differences between populations may result in variable risk factors, an approach that includes traditional CRA methodology as well as microbiological based predictive tools may provide an improved individualized assessment and better preventive treatment plan. This could include not only identifying those most at risk, but also those least at risk for caries development in areas where access to care is limited.

## 5. Limitations

Studies of microbial flora are limited by study methodology. In this study, we chose to use selective agars to obtain the targeted microbial counts. The use of selective media can result in undercounts. Other factors that can affect accuracy include mechanical (plating volume inaccuracies during spiral plating or slight variations in commercial media preparations) and manual influences (poor sample quality, incomplete mixing of the sample before diluting and plating or counting errors). These limitations were additional factors considered in the decision to express the data as proportions rather than absolute counts.

The largest challenge in this study was missed visits, which limited the data available for analysis. Enrollment began in December of 2018. In the spring of 2020, all clinical research activities were shut down for six months due to the emerging pandemic. This resulted in not being able to collect samples from any subjects whose sample window fell within that time period and prevented enrollment for the duration of the shut down as well. The areas most impacted by the temporary halt of the study were enrollment, 6-month and 12-month follow-up visits due to be scheduled during that time. In order to mitigate the effects of missed visits on statistical analyses in this study, pairs were matched that had the same visit data available (i.e., both subjects had baseline, 12-months and 18-month samples, but no 6-month sample due to the shutdown). The missed visits were due to circumstances beyond our control, but ultimately resulted in gaps in the data leading up to caries diagnosis for four subject pairs at 6 months before caries onset and three pairs at 12 months before caries onset. The number of subject pairs available for analysis in this study was also limited by the number of children who have completed the study and developed caries over the course of the 18 months. As additional children exit the study after their final visit, we anticipate being able to expand these analyses to include more pairs of CA/CF children.

## 6. Conclusions

In this subset of children selected from our larger longitudinal study, we observed multiple microbiological measures that were associated with the development of dental caries. When setting a cut-off value to indicate higher disease burden, acid tolerant bacteria, mutans streptococci, and *Candida* species were all associated with eventual ECC development. Co-colonization of MS and *Candida* was also strongly associated with the onset of caries. While ECC is a multifactorial disease with many recognized risk factors, there is clear value in identifying microbiological conditions that can reliably be evaluated in order to more accurately assess ECC risk and potentially mark the start of the shift to oral microbiome dysbiosis. This could lead to new predictive methods and novel approaches to disrupt the initiation and progression of the disease before it is clinically evident.

As work on the overarching longitudinal study continues, future studies will expand existing analyses to additional pairs of CA/CF children within the study population. Additionally, the role of non-mutans low pH streptococci in caries development in these children will be explored, which could further increase the feasibility of using this combination of microbiological factors in predicting future caries.

## Data Availability

The raw data supporting the conclusions of this article will be made available by the authors, without undue reservation.
